# Defective Monocyte Enzymatic Function and an Inhibitory Immune Phenotype in Human Immunodeficiency Virus-Exposed Uninfected African Infants in the Era of Antiretroviral Therapy^[Author-notes jiac133-FM1]^

**DOI:** 10.1093/infdis/jiac133

**Published:** 2022-04-11

**Authors:** Louise Afran, Kondwani C Jambo, Wilfred Nedi, David J C Miles, Anmol Kiran, Dominic H Banda, Ralph Kamg’ona, Dumizulu Tembo, Annette Pachnio, Eleni Nastouli, Brigit Ferne, Henry C Mwandumba, Paul Moss, David Goldblatt, Sarah Rowland-Jones, Adam Finn, Robert S Heyderman

**Affiliations:** Malawi-Liverpool-Wellcome Trust Clinical Research Programme, University of Malawi College of Medicine, Blantyre, Malawi; Bristol Children’s Vaccine Centre, Schools of Cellular & Molecular Medicine and of Population Health Sciences, University of Bristol, Bristol, United Kingdom; Department of Clinical Sciences, Liverpool School of Tropical Medicine, Liverpool, United Kingdom; Malawi-Liverpool-Wellcome Trust Clinical Research Programme, University of Malawi College of Medicine, Blantyre, Malawi; Department of Clinical Sciences, Liverpool School of Tropical Medicine, Liverpool, United Kingdom; Malawi-Liverpool-Wellcome Trust Clinical Research Programme, University of Malawi College of Medicine, Blantyre, Malawi; Malawi-Liverpool-Wellcome Trust Clinical Research Programme, University of Malawi College of Medicine, Blantyre, Malawi; Institute of Immunology and Immunotherapy, University of Birmingham, Cancer Sciences Building, Edgbaston, Birmingham B15 2TT, United Kingdom; Malawi-Liverpool-Wellcome Trust Clinical Research Programme, University of Malawi College of Medicine, Blantyre, Malawi; Center for Inflammation Research, Queens Research Institute, University of Edinburgh, Edinburgh, United Kingdom; Malawi-Liverpool-Wellcome Trust Clinical Research Programme, University of Malawi College of Medicine, Blantyre, Malawi; Malawi-Liverpool-Wellcome Trust Clinical Research Programme, University of Malawi College of Medicine, Blantyre, Malawi; Malawi-Liverpool-Wellcome Trust Clinical Research Programme, University of Malawi College of Medicine, Blantyre, Malawi; Institute of Immunology and Immunotherapy, University of Birmingham, Cancer Sciences Building, Edgbaston, Birmingham B15 2TT, United Kingdom; Division of Infection and Immunity, University College London, London, United Kingdom; Division of Infection and Immunity, University College London, London, United Kingdom; Malawi-Liverpool-Wellcome Trust Clinical Research Programme, University of Malawi College of Medicine, Blantyre, Malawi; Department of Clinical Sciences, Liverpool School of Tropical Medicine, Liverpool, United Kingdom; Institute of Immunology and Immunotherapy, University of Birmingham, Cancer Sciences Building, Edgbaston, Birmingham B15 2TT, United Kingdom; Division of Infection and Immunity, University College London, London, United Kingdom; Nuffield Department of Medicine, University of Oxford, Oxford, United Kingdom; Malawi-Liverpool-Wellcome Trust Clinical Research Programme, University of Malawi College of Medicine, Blantyre, Malawi; Malawi-Liverpool-Wellcome Trust Clinical Research Programme, University of Malawi College of Medicine, Blantyre, Malawi; Division of Infection and Immunity, University College London, London, United Kingdom

**Keywords:** HIV-exposed uninfected, human herpes virus', vaccine responses, Heamophilus influenzae type b, monocytes

## Abstract

**Background:**

Human immunodeficiency virus-exposed uninfected (HEU) infants are a rapidly expanding population in sub-Saharan Africa and are highly susceptible to encapsulated bacterial disease in the first year of life. The mechanism of this increased risk is still poorly understood. We investigated whether human immunodeficiency virus (HIV)-exposure dysregulates HEU immunity, vaccine-antibody production, and human herpes virus amplify this effect.

**Methods:**

Thirty-four HIV-infected and 44 HIV-uninfected pregnant women were recruited into the birth cohort and observed up to 6 weeks of age; and then a subsequent 43 HIV-infected and 61 HIV-uninfected mother-infant pairs were recruited into a longitudinal infant cohort at either: 5–7 to 14–15; or 14–15 to 18–23 weeks of age. We compared monocyte function, innate and adaptive immune cell phenotype, and vaccine-induced antibody responses between HEU and HIV-unexposed uninfected (HU) infants.

**Results:**

We demonstrate (1) altered monocyte phagosomal function and B-cell subset homeostasis and (2) lower vaccine-induced anti-*Haemophilus influenzae* type b (*Hib)* and anti-tetanus toxoid immunoglobulin G titers in HEU compared with HU infants. Human herpes virus infection was similar between HEU and HU infants.

**Conclusions:**

In the era of antiretroviral therapy-mediated viral suppression, HIV exposure may dysregulate monocyte and B-cell function, during the vulnerable period of immune maturation. This may contribute to the high rates of invasive bacterial disease and pneumonia in HEU infants.

## Teaser

Early life HIV exposure may dysregulate innate and adaptive immunity, specifically, monocyte function and vaccine-induced immunity to encapsulated bacteria. This may lead to altered protection and susceptibility to disease from encapsulated bacteria.

Human immunodeficiency virus-exposed uninfected (HEU) infants are particularly vulnerable to invasive bacterial disease [1, 2], particularly, pneumonia [3] and diarrhea [4], and they have more frequent hospitalizations, more severe infections, and increased risk of treatment failure. However, the mechanism of this increased vulnerability remains unknown. The global population of HEU children is substantial, estimated at 1.2 million births annually, mainly within developing countries [5]. Therefore, a coordinated strategy is necessary to ensure their optimal health and wellbeing [6].

The vulnerability of HEU infants is likely a complex intersection of human immunodeficiency virus (HIV)-exposure immune profile “remodeling”, “inflammatory” maternal cytokine milieu [7], time of antiretroviral therapy (ART) initiation [8], ART use [9] and prophylactics [10], increased exposure to maternal viral/bacterial pathogens, and host microbial/environmental factors. Prevaccination antibody levels are lower in HEU infants, but they are normalize to HIV-unexposed uninfected (HU) infants’ levels postvaccination [11–13]. Taken together, these result in a more permissive state for the development of infections [1]. Many observational studies reported immunological abnormalities in HEU infants [14] including highly differentiated T-cell [15] and B-cell subsets [16], altered responses to vaccines [17], functional impairment of natural killer cells [18], and monocytes [19]. Furthermore, few studies considered early transmission of immunomodulatory human herpes virus (HHV), cytomegalovirus (CMV) [20], and/or Epstein-Barr virus (EBV) [21] recrudescence during pregnancy, because HHVs are important drivers of inflammation in HEU infants [20].

Due to the successful HIV test and treatment strategy globally, the prevalence of individuals receiving ART has risen considerably [5]. As a result, the number of HEU infants born to mothers receiving ART increased markedly; however, despite expanded implementation of prevention of vertical transmission programs, the risk of infection-related morbidity and mortality among HEU infants remains high [2], particularly the risk of encapsulated bacterial infection [22]. The immune profile among HEU infants is not well documented. Therefore, we addressed whether HIV-exposure and HHVs dysregulate infant immunity and/or the response to primary vaccination.

## METHODS

### Study Design and Population

The study was conducted in Southern Malawi, at Ndirande Health Centre (primary healthcare facility in Blantyre) and at Queen Elizabeth Central Hospital (QECH) (tertiary teaching hospital in Blantyre). We recruited HEU and HU infant cohorts in 2 contiguous groups that were followed longitudinally pre- and postroutine childhood vaccination with pentavalent DPT-HepB-Hib immunization. The first group were aged 5–9 weeks (pre-1st vaccine dose), who were followed up to age 14–15 weeks (post-2nd vaccine dose), and the second group were aged 14–15 weeks (post-2nd vaccine dose), who were followed up to 18–23 weeks of age (post-3rd vaccine dose). We also recruited pregnant women in the early stages of labor at QECH maternity ward and subsequently their babies at birth (termed newborn birth cohort). Participating mothers were healthy (without disease), asymptomatic adults (≥18 years) comprising HIV-infected and HIV-uninfected volunteers. Human immunodeficiency virus testing was performed on maternal whole blood in the early stages of labor or at recruitment using 2 commercial point-of-care rapid HIV test kits, Determine HIV 1/2 kit (Abbott Diagnostic Division, Abbott Park, IL) and Unigold HIV 1/2 kit (Trinity Biotech Inc., Bray, Ireland), and had a CD4^+^ T-cell count performed. Human immunodeficiency virus-infected participants received first-line ART (Option B+ [Lamivudine, Tenofovir DF, and Efavirenz {3TC/TDF/EFV}]) during or prepregnancy. All HEU babies received nevirapine at birth for 6 weeks, followed by co-trimoxazole until 1 year of life. Exclusion criteria for the study participants were current or past history of smoking, heart disease, tuberculosis (TB), high blood pressure, drug use, syphilis, severe anemia (hemoglobin <8 g/dL), placental abnormalities, infant prematurity, low birth weight or death, and existing comorbidities. All babies received Bacille Calmette-Guerin (BCG) vaccine at birth. Written informed consent was obtained from all participants before recruitment. Ethical approval was obtained from the University of Malawi College of Medicine Research and Ethics Committee (COMREC) (Blantyre, Malawi; protocol numbers P.11/11/1140, P.06/11/1088).

### Sample Collection and Processing

We collected 5 mL venous blood from the infants, at 5–9, 14–15, and 18–23 weeks of age after attendance at the vaccination clinic for pentavalent DPT-HepB-Hib immunization. Participants with incomplete vaccine course were excluded. Mothers had 10 mL venous blood collected at the first visit and 3 mL breastmilk collected at all other time points. We collected up to 40 mL cord blood from the umbilical vein into sodium heparinized tubes using a 50-mL syringe, from the newborn interface of the placenta immediately after birth. Whole blood was kept at room temperature and processed within 2 hours. Peripheral blood mononuclear cells were isolated by density centrifugation. Plasma was separated by centrifugation at 1500 rpm for 10 minutes, aspirated, aliquoted, and stored at −80°C for later use. Breastmilk samples were collected by hand expression, fractionated into lipid and aqueous phase, and stored at −80°C. Due to limitations in the volume of blood collectable from very young babies and limited cell numbers, not all the assays were performed on every newborn or infant sample.

### Human Immunodeficiency Virus Testing

Newborns’ and infants’ qualitative HIV deoxyribonucleic acid (DNA) polymerase chain reaction (PCR) tests were performed in batches of 23 at Malawi Liverpool Wellcome (MLW) core laboratories. Total DNA was isolated from 0.5 × 10^6^ cells using AMPLICOR HIV-1 DNA test, V1.5 (Roche, Brea, USA)) according to the manufacturer’s instructions. Human immunodeficiency virus DNA results in participant health passports were also collected.

Three HIV-RDT kits, from 2 separate manufacturers (Unigold and Determine), were used to confirm the presence or absence of HIV-specific immunoglobulins (Igs) in maternal peripheral blood. Cord blood mononuclear cells were stored in 500 µL RNAlater and analyzed at University College London (UCL) by digital PCR as described elsewhere [23].

### Maternal CD4 Counts

Peripheral blood CD4 T-counts and full blood count were performed at the MLW Trust Clinical Research Programme Diagnostic Laboratory on an HmX analyzer (Beckman Coulter, Indiana, USA) using a standardized protocol. Blood was taken in the early stages of labor.

### Phenotypic Analysis

Multicolor flow cytometry analysis was performed on whole blood. Samples were stained with the following fluorochrome-conjugated antibodies: anti-CD14 Phycoerythrin Cyanine-7 (PECy7), anti-CD3 Allophycocyanin-H7 (APC-H7), anti-CD4 Pacific Blue (PB), anti-CD8 Fluorescein (FITC), anti-CD8 (PECY7), CD45RA Phycoerythrin (PE), CD45RA PECY5, CCR7 Allophycocyanin (APC), anti-CD19 (APC), anti-CD19 Peridinin-Chlorophyll-Protein (PERCP), anti-CD27 (PE), CD10 PE-Cy7, CD21 FITC, CD27 APC-Cy7, CD95 e450, FcLR4 PE, CD57 FITC, PD-1 APC, and PD-1 PE (Supplementary 2–5 Gating Strategy and Table 2). Samples were acquired on Beckman Coulter Cyan ADP and analyses were performed using FlowJo Version 7.6.5 and 10.5 software (TreeStar).

### Measurement of Monocyte Phagosomal Enzymatic Activity

Phagosomal oxidative burst and bulk proteolytic function in monocytes was measured using a flow cytometry-based reporter bead assay as described previously [24] (Supplementary Methods 1A).

### T-Cell Interferon-γ Enzyme-Linked Immunospot

A total of 2 × 10^6^ peripheral blood mononuclear cells (PBMCs) were stimulated with GAG peptides in an 18-hour interferon (IFN)γ T-cell enzyme-linked immunospot (ELISpot) as previously described [25] (Supplementary Methods 1B).

### Human Cytomegalovirus Polymerase Chain Reaction

Real-time PCR was used to detect human CMV (hCMV) in HIV-infected and uninfected maternal breast milk and infant oropharyngeal throat swabs (Supplementary Methods 1C).

### Detection of Cytomegalovirus-Specific Immunoglobulin (Ig)G and IgM Antibodies

Human CMV-specific IgM in HIV-infected and uninfected maternal and infant plasma was measured using a commercial enzyme-linked immunosorbent assay (ELISA) kit (IBL International, Hamburg, Germany) according to manufacturer’s instructions. A semiquantitative, in-house hCMV IgG assay was used at the laboratories in the University of Birmingham, United Kingdom (Supplementary Methods 1D).

### Epstein-Barr Virus Nested Polymerase Chain Reaction

A nested PCR measuring EBNA3B gene in HIV-infected and uninfected maternal breast milk and infant throat swabs were used (method as described in [26]).

### Detection of Epstein-Barr Virus-Specific Immunoglobulin G Antibodies

Immunoglobulin G antibodies against EBV viral capsid antigen (VCA) were detected in plasma using a commercial ELISA kit (Diagnostic Automation, Woodland Hills, CA) according to the manufacturer’s instructions.

### Sandwich Enzyme-Linked Immunosorbent Assay to Detect Immunoglobulin G Specific to Vaccine Antigens

In an in-house ELISA, tetanus toxoid (TT) or diphtheria toxoid (DT) (both National Institute for Biological Standards and Control [NIBSC] were tested, Potters bar, UK) (Supplementary Methods 1E). Optical density was measured (without acid stopping the reaction) after 10 minutes using an ELISA plate reader (Biotek, Cheshire, UK) set at 405 nm and SoftMax Pro software.

### Multiplexed Opsonophagocytosis Killing Assay and Serotype-Specific Immunoglobulin G

Immunoglobulin G serum concentrations specific for the 13 pneumococcal vaccine serotypes (1, 3, 4, 5, 6 A, 6B, 7F, 9 V, 14, 18C, 19A, 19F, and 23F) were measured using an ELISA (described in Supplementary Methods 1F).

### Statistical Analysis

Statistical analysis and graphical presentation were performed using Prism 7/8 (GraphPad Software, San Diego, CA), and Python (Python Software Foundation) was used to calculate summary statistics. Demographic and clinical characteristics were compared using Mann-Whitney *U* tests for continuous and Fisher’s exact tests or χ^2^ for discrete variables. The ELISpot data were reported as subtracted 2× background. Serotype-specific opsonophagocytic indexes (OPIs) were reported using geometric means and 95% confidence intervals. The OPIs were classified as being positive or negative based on the current recommended cutoff value of <8 (negative) and ≥8 (positive). Results are reported as median and interquartile range (IQR) as stated.

## RESULTS

### Participant Characteristics

In the newborn birth cohort, 34 HIV-infected and 44 HIV-uninfected pregnant women were recruited: 2 newborns were excluded from the analysis due to death and HIV-positivity detected by digital droplet PCR. Human immunodeficiency virus-infected pregnant women received ART Option B+ (tenofovir/lamivudine/efavirenz) for an average of 18.7 (range, 1–143) months, with a mean nadir CD4^+^ T-cell count of 294 (range, 8–892) and were more likely to have had an elective caesarean birth, compared with HIV-uninfected pregnant women (*P* = .01). In the longitudinal infant cohort, 43 HIV-infected and 61 HIV-uninfected mother-infant pairs were recruited and sampled across time points, 5–9, 14–15, 18–23 weeks of age, corresponding to the Malawian routine infant vaccine schedule of the following: BCG at birth, then pentavalent vaccine at 6, 10, 14 weeks. Human immunodeficiency virus-infected women had received option B+ for an average of 9.28 (range, 1–72) months at the time of enrollment and had a mean nadir CD4^+^ T-cell count of 409 (range, 159–823). There was no difference between maternal age or breastfeeding status, but mode of delivery was more often caesarean section in HIV-infected mothers compared with HIV-negative mothers (Table 1).

**Table 1.** Participant Characteristics Among HEU Newborns, Infants, and HU Controls

	n = 44	n = 34		n = 61	n = 43	
	Newborn Birth Cohort			Longitudinal Infant Cohort		
Maternal Status	HIV^−^	HIV^+^	*P* Value	HIV^−^	HIV^+^	*P* Value
Mothers age in years median (IQR)^a^	27.6 (23.6–32.95)	29.7 (25.95–32.2)	.86	21.9 (19.5–26.2)	28.8 (25.2–33.1)	.37
Mothers ART no. (%)^a^	N/A	34/34 (100)	…	N/A	37/43 (86)	…
Mothers time on ART, months, mean (range)^a^	N/A	18.17 (1–143)	…	N/A	9.28 (0–72)	…
Mothers time on ART, prepregnancy count (%)^a^	N/A	16/34 (47)		N/A	10/43 (23)	
Mothers time on ART, 1st trimester count (%)^a^	N/A	0/34 (0)		N/A	1/43 (2)	
Mothers time on ART, 2nd trimester count (%)^a^	N/A	9/34 (26)		N/A	5/43 (12)	
Mothers time on ART, 3rd trimester count (%)^a^	N/A	9/34 (26)		N/A	15/43 (35)	
Mothers who did not receive ART during pregnancy, count (%)^a^	N/A	0/34 (0)		N/A	12/43 (28)	
Mothers CD4^+^ cells µL mean (range)^a^	N/A	294 (8–892)	N/A	N/A	409 (159–823)^b^	…
Mothers, no. caesarean Section (%)^a^	0/44 (0)	14/34 (39)	**.01**	…	…	…
No. mothers self-reported exclusive breastfeeding (%)	44/44 (100)	34/34 (100)	…	61/61	43/43	…
No. female Sexed children (%)^a^	22/44	15/34	.65	29/61 (55)	22/43 (39)	.24
No. who tested negative for HIV DNA PCR at birth (%)	…	31/34 (100)		…	43/43	…
No. who tested negative for HIV DNA PCR at 6 weeks (%)	…	34/34 (100)		…	36/43 (83.7)^c^	…

Abbreviations: ART, antiretroviral therapy; DNA, deoxyribonucleic acid; dPCR, digital polymerase chain reaction; HEU, human immunodeficiency virus-exposed uninfected; HIV, human immunodeficiency virus; HU, HIV-unexposed uninfected; IQR, interquartile range; N/A, not applicable; PCR, polymerase chain reaction; RDT, rapid diagnostic test performed on whole blood.

NOTE: HIV-infected participants received first-line ART (Option B+ [3TC/TDF/EFV]) at any point during pregnancy and had a CD4^+^ T-cell count performed. Newborns and infants were tested with an HIV DNA PCR test at birth and 6 weeks as part of the early infant diagnosis program in Malawi, and newborns were additionally tested by digital droplet HIV DNA PCR at University College London laboratories. Mothers self-reported exclusive breast feeding at the time of recruitment, which was birth, 6 weeks, and 14 weeks. Significant figures *P* = < 0.05 are shown in bold.

a Calculated using Mann-Whitney *U* test for continuous variables or Fisher’s exact test for categorical variables.

b Three mothers had missing CD4 data. Median (IQR) and mean (range) are reported.

c Seven of forty-three unconfirmed HIV status.

### Defective Monocyte Enzymatic Function

**Figure 1.**
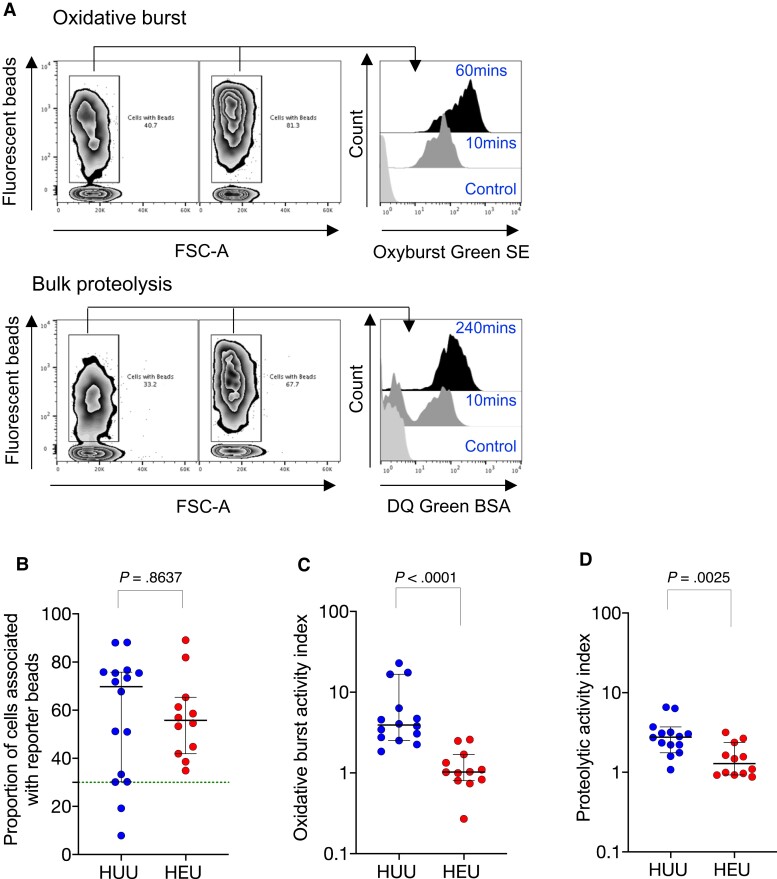
Monocyte phagosomal functional capacity in human immunodeficiency virus-exposed uninfected (HEU) newborns and human immunodeficiency virus-unexposed uninfected (HU) controls. The proportion of CD14^+^ cells that performed (*A*) phagosomal superoxide burst activity and phagosomal bulk proteolytic activity (*B*) and that were associated with beads, (*C*) the phagosomal superoxide burst activity index, and (*D*) the phagosomal bulk proteolytic activity index. The readout for the assay is reported as the median fluorescent intensity of the reporter fluorochrome at 60 minutes (mins):10 mins and 240 mins:10 mins for oxidative burst and bulk proteolysis, respectively. The activity index was calculated using a ratio of the reporter fluorochrome over the calibration fluorochrome. Only individuals with an uptake of greater than ≥30% were used in the phagosomal analysis. Data are presented as medians (interquartile range) and analyzed using Mann-Whitney *U* test (HU, n = 16; HEU, n = 12). BSA, bovine serum albumin; FSC, forward scatter.

First, we used a flow cytometry-based whole blood phagocyte functional reporter bead assay [24] to assess monocyte function in cord blood from the birth cohort (Figure 1A; Supplementary Figure 1). We assessed the ability of monocytes to internalize Alexa Fluor 405-labeled IgG-coated reporter beads at 1 hour post coincubation, as a proxy of uptake capacity. We showed that the proportion of monocytes that internalized reporter beads was similar between HEU infants and HU controls (56 [IQR, 43–64] vs 70 [IQR, 31–76]; *P* = .86) (Figure 1B) (HU, n = 16; HEU, n = 12). We next assessed the phagosomal superoxide burst activity, and we found that it was lower in monocytes from HEU infants compared with HU controls (1 [IQR, 0.8–1.6] vs 3.9 [IQR, 2.7–8.9]; *P* = .0001) (Figure 1C). Third, we assessed the phagosomal bulk proteolytic activity and showed that it was lower in monocytes from HEU newborns compared with HU controls (1.3 [IQR, 0.94–2.2] vs 2.8 [IQR, 2.1–3.3]; *P* = .0025) (Figure 1D). Taken together, these data indicate altered monocyte phagosomal functional capacity at birth in HEU newborns.

### Increased B-Cell Inhibitory Phenotype and Purified Protein Derivative Responses but Decreased T-Cell Phytohemagglutinin Responses

**Figure 2.**
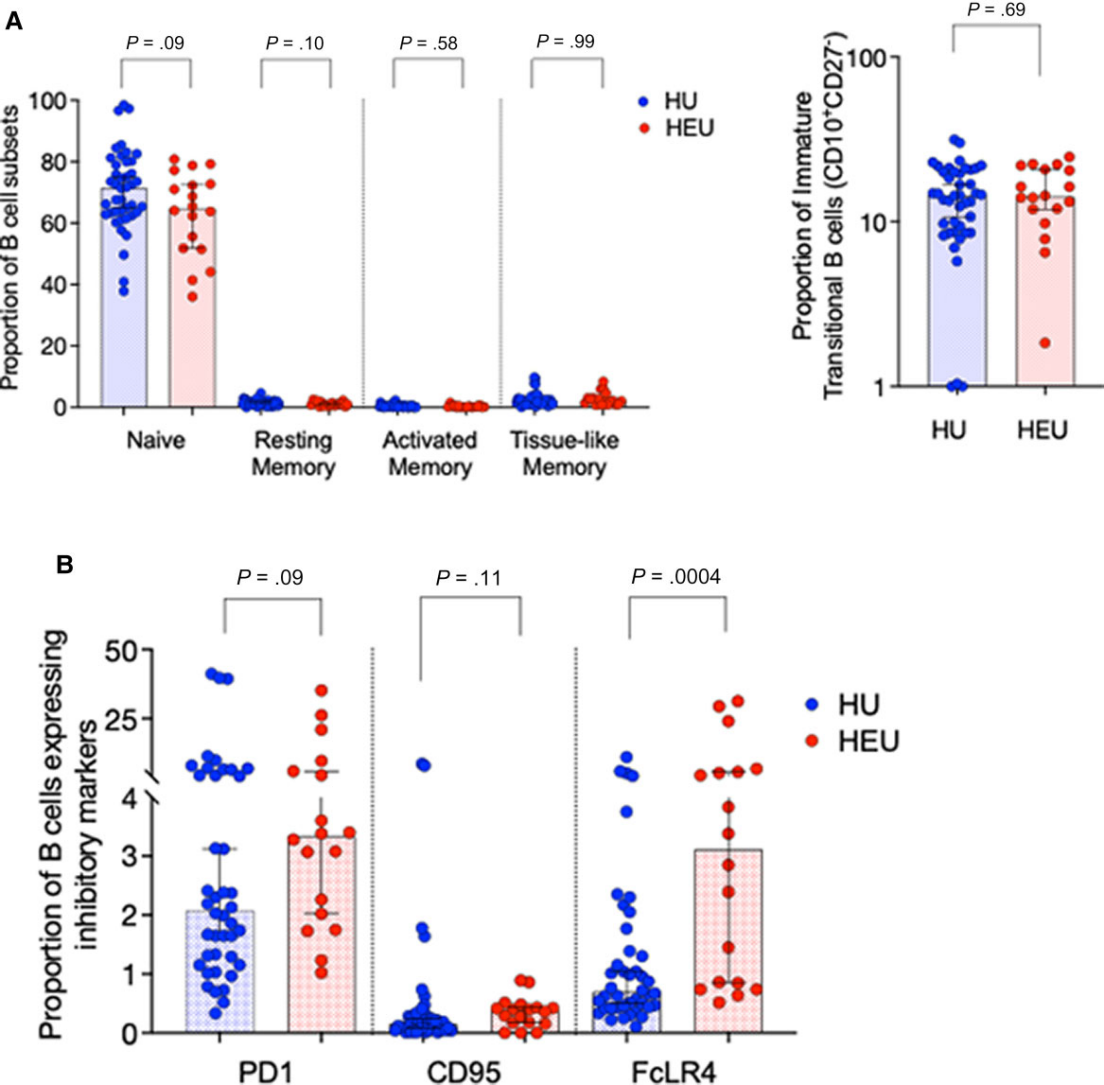
Characterization of B-cell immune profiles in human immunodeficiency virus-exposed uninfected (HEU) and human immunodeficiency virus-unexposed uninfected (HU) newborns. Cord blood was stained with the following fluorochrome-conjugated antibodies: anti-CD19 APC, anti-CD10 PE-Cy7, anti-CD21-FITC, and anti-CD27 APC-CY7. Singlets were defined using forward scatter (FSC)-A vs FSC-H parameters, and lymphocytes were gated using side scatter (SSC)-A and FSC-A. B cells were then gated using CD19 against SSC-A. (*A*) The proportion of B-cell subsets were clasified using CD10, CD21, and CD27 as follows: CD10^−^CD21^+^CD27^−^ (naive), CD10^−^CD21^−^CD27^+^ (resting memory), CD10^−^CD21^+^CD27^+^ (activated memory), CD10^−^CD21^−^CD27^−^ (tissue-like memory), and CD10^+^CD27^−^ (immature transitional). (*B*) Expression of CD95e450, FcLR4 PE, and PD-1 APC (exhausted and activatory inhibited B cells) were gated as a proportion of CD19^+^ cells. Data are presented as medians (interquartile range) and analyzed using Mann-Whitney *U* test (HU n = 42, HEU n = 18 B cells; and HU n = 41; HEU n = 24 T cells).

We next sought to investigate dysregulation in the adaptive arm of immunity using cord blood. We observed similar distributions of B-cell subsets between HEU and HU newborn babies (*P* > .1), including, CD10^−^CD21^+^CD27^−^ (naive), CD10^−^CD21^−^CD27^+^ (resting memory), CD10^−^CD21^+^CD27^+^ (activated memory), CD10^−^CD21^−^CD27^−^ (tissue-like memory), and CD10^+^CD27^−^ (immature transitional) B cells (Figure 2A) (HU, n = 42; HEU, n = 18). However, we found a higher proportion (percentage) of Fc-receptor like 4 (FcRL4^+^) expressing B cells in HEU newborns (3.11 [IQR, 0.82–6] vs 0.7 [IQR, 0.44–1.5]; *P* = .0004) but no statistically significant differences in the proportion of PD-1 (3.3 [IQR, 2–6.9] vs 2.1 [1.3–5]; *P* = .09) or CD95-expressing B cells (0.38 [IQR, 0.17–0.44] vs 0.15 [IQR, 0.05–0.31]; *P* = 0.1), when compared with HU controls (Figure 2B). FcLR4^+^ inhibits B-cell activation through the B-cell receptor (BCR) and is a marker of B-cell exhaustion in chronically HIV-infected adults, which suggests increased B-cell regulation in HEU newborn babies.

Next, using peripheral blood collected from the longitudinal infant cohort aged 5–9 weeks, we also found B-cell alterations in 2 subsets that are selectively dysregulated during chronic HIV infection [27, 28]. The proportions of immature transitional and tissue-like memory B cells were lower in HEU infants than HU controls (7.6 [IQR, 1.2–16] vs 12 [IQR, 6.4–21]; *P* = .04) (Supplementary Figure 2A); however, the proportions of naive and central memory, CD4^+^ and CD8^+^ T-cell subsets were similar ( *P* > .1) (Supplementary Figure 2B and C).

**Figure 3.**
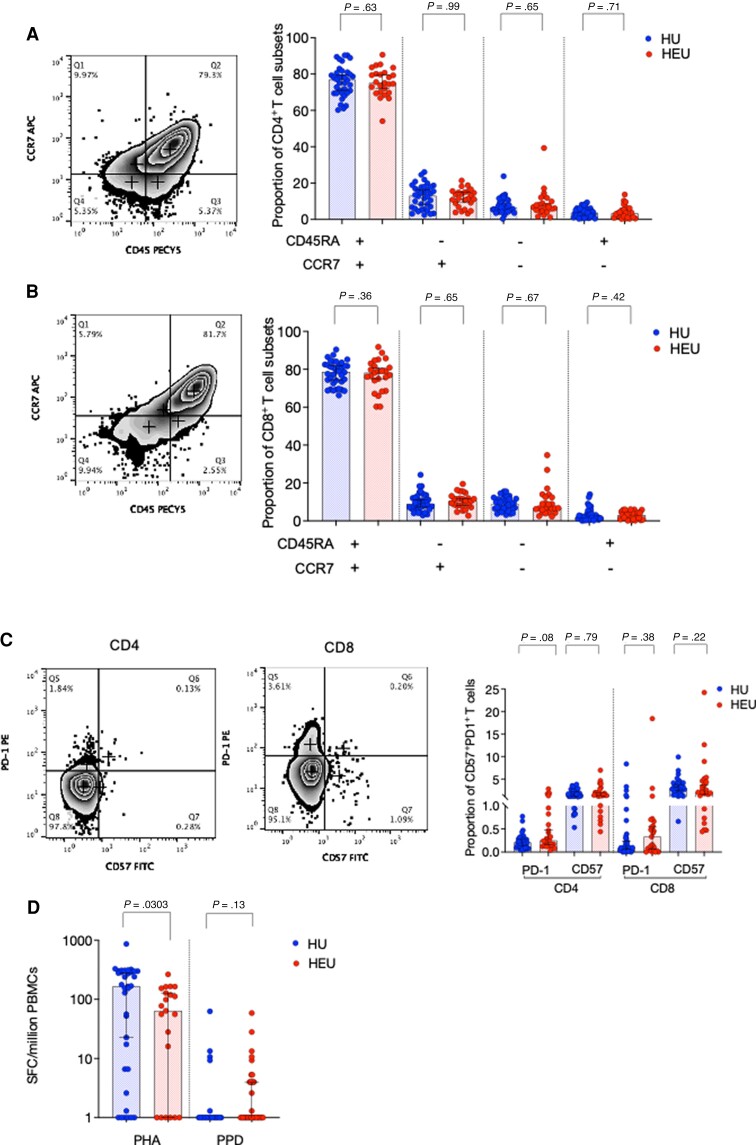
Characterization of T-cell subsets in human immunodeficiency virus-exposed uninfected (HEU) and human immunodeficiency virus-unexposed uninfected (HU) newborns. Whole cord blood was stained with the following fluorochrome-conjugated antibodies: anti-CD3 APCCY7, anti-CD4 Pacific Blue, anti-CD8-FITC, anti-CCR7 APC, and anti-CD45RA. PECY7 were used to identify T-cell subsets. Singlets were defined using forward scatter (FSC)-A vs FSC-H parameters, and lymphocytes were gated using side scatter (SSC)-A and FSC-A. T cells were then gated using CD3 against SSC-A, then a CD4 versus CD8 plot was used to separate the 2 main T-cell subsets. A second panel of fluorochrome-conjugated antibodies—anti-CD3 APCCY7, anti-CD4 Pacific Blue, anti-CD8-PECY7, CD57 FITC, and PD1-PE—were used to measure T-cell senescence and exhaustion (HU, n = 36; HEU, n = 25). (*A*) CD4^+^ and (*B*) CD8^+^ T-cell subsets were clasified using CCR7 and CD45RA as follows: CCR7^−^CD45RA^−^ (effector memory), CCR7^+^CD45RA^−^ (central memory), CCR7^+^CD45RA^+^ (naive), and CCR7^−^CD45RA^+^ (terminally differentiated). (*C*) CD4^+^ and CD8^+^ T-cell subsets were clasified using CD57 (senescent) and PD-1 (exhausted) expression. (*D*) Isolated cord blood mononuclear cells were incubated with phytohemagglutinin (PHA) or purified protein derivative (PPD) for 18 hours, and interferon-γ-producing cells were detected on a 96-well microtiter enzyme-linked immunospot plate. The frequency of spot-forming cells (SFCs)/million cord blood mononuclear cells are plotted for all subjects (HU, n = 35; HEU, n = 21). Data were analyzed using Fisher’s exact test. Error bars depict medians (95% confidence interval).

When we looked at T cells from cord blood, the proportion of naive and central memory CD4^+^ and CD8^+^ T-cell subsets measured by CD45RA-CCR7 expression were similar between HEU newborns and HU controls (*P* > .5 and *P* > .1, respectively) (Figure 3A and B) (HU, n = 41; HEU, n = 24), as were the proportion of CD57 or PD-1-expressing CD4^+^ (*P* = .08, *P* = .79) and CD8^+^ T cells (*P* = .38, *P* = .22), respectively (Figure 3C). Moreover, mean IFNγ production in response to tuberculin purified protein derivative (PPD) in an 18-hour ELISpot assay was similar in HEU newborns compared with controls (1 [1–62] vs 1[1–58] [min-max]; *P* = .13) (HU, n = 35; HEU, n = 21). In this population, BCG is received soon after birth. However, IFNγ production in response to the selective T-cell mitogen phytohemagglutinin (PHA) was reduced in HEU newborns compared with controls (64 [1–141] vs 165 [6.6–299]; *P* = .03) (Figure 3D), indicating selective regulation of T-cell responses. We then evaluated PBMCs from infant peripheral blood, for IFNγ spot-forming cells (SFCs) to TT, hepatitis B (Hb), and PPD in an 18-hour T-cell ELIspot. We observed increased IFNγ SFCs/million PBMCs to PPD among HEU infants, compared with HU controls (143 [42–268] vs 34 [3.3–79]; *P* = .03), but similar Hb (*P* = .78), TT (*P* = .26), and PHA (*P* = .75) responses (Supplementary Figure 2D). These data indicate that antigen-specific responses to PPD in HEU infants after BCG vaccination are enhanced.

### Decreased Vaccine-Induced Anti-Hemophilus Influenzae Type b and Diphtheria Toxoid Titers but Preserved Opsonophagocytosis Activity to Pneumococcal Conjugate Vaccine 13 Serotypes

Using peripheral blood from infants, we next interrogated vaccine-induced memory B-cell antibody responses to polysaccharide and protein antigens that are in the Malawian infant primary vaccination series; including pneumococcal conjugate vaccine (PCV)13 (HU, n = 11; HEU, n = 9) and pentavalent vaccine (DPT-HepB-Hib) (HU, n = 50; HEU n = 39), 14–15 (HU, n = 22; HEU, n = 27) and 18–23 (HU, n = 25; HEU, n = 19).

**Figure 4.**
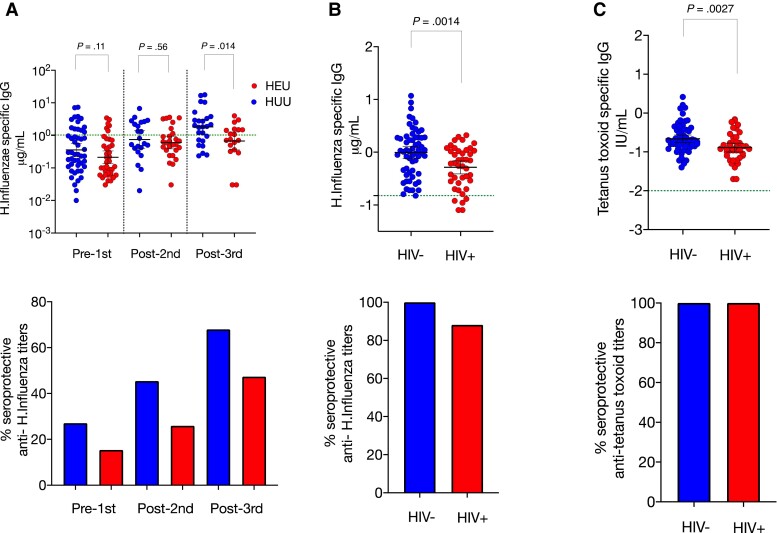
Infant and maternal antibody responses. Preceding and after Penta-DTwPHibHepB vaccination, we measured vaccine titers using an enzyme-linked immunosorbent assay to (*A*) anti-Hib immunoglobulin G (IgG) in infant serum at 5–7 (human immunodeficiency virus-unexposed uninfected [HU], n = 50; human immunodeficiency virus-exposed uninfected [HEU], n = 39), 14–15 (HU, n = 22; HEU, n = 27), and 18–23 (HU, n = 25; HEU, n = 19) weeks; we also measured (*B*) maternal anti-*Hib* IgG (*C*) maternal anti-TT IgG at 1 timepoint in human immunodeficiency virus (HIV)-uninfected (n = 61) and HIV-infected (n = 43). Blue circles are controls and red are HEU infants or HIV-infected mothers. Green dotted horizontal line represents cutoff for protective titers. Data are presented as mean (95% confidence interval) and analyzed using Mann-Whitney *U* test. Minimum putative protective titers are 0.15 µg/mL (passive) and 1.0 µg/mL (acquired) for *Hib* [30] and 0.01 IU/mL for tetanus toxoid and diphtheria toxoid.

After 3 vaccine doses, we found lower geometric mean titer anti-*Hib* (0.67 [standard deviation {SD} = 3.7] vs 1.8 [SD = 3.4]; *P* = .014) and anti-DT titers (1.6 [SD = 7.4] vs 4.1 [SD = 2.2]; *P* = .036), but similar anti-TT titers (0.47 [SD = 0.4] vs 0.57 [SD = 0.28]; *P* = .27) in HEU infants, compared with HU controls (Figure 4A and Supplementary Figure 6A and B). In the mothers of these infants, anti-*Hib* IgG (0.52 [SD = 2.5] vs 0.99 [SD = 2.8]; *P* = .0015) and anti-TT (0.13 [SD = 2.3] vs 0.21 [SD = 2.4]; *P* = .002) IgG titers but not anti-DT titers (0.05 [SD = 4.62] vs 0.05 [SD = 3.5]; *P* = .91) were lower in HIV-infected mothers than HIV-uninfected controls (Figure 4B and C and Supplementary Figure 6C).

We next tested whether HIV exposure influences the levels of vaccine-induced functional antibody in infants aged 5–9 weeks. Using a Multiplex Opsonophagocytosis Assay, we measured opsonophagocytic activity of 13 vaccine serotypes from the PCV13 (1, 3, 4, 5, 6A, 6B, 7F, 9V, 14, 18C, 19A, 19F, 23F) in infant sera. We found no difference in the geometric mean OPI of 13 pneumococcal serotypes and geometric mean concentration (GMC) of serotype-specific IgG titers between HEU infants and HU controls (Table 2).

**Table 2.** Robust Opsonizing and Killing Function of Anti-Pneumococcal Capsular Polysaccharide-Specific IgG Antibodies in Infant Serum^a^

	n = 11	n = 9		n = 11	n = 8	
PCV13 Serotypes	HU Infants GMOI (95% CI)	HEU Infants GMOI (95% CI)	*P* Value	HU Infants GMC (95% CI)	HEU Infants GMC (95% CI)	*P* Value
4	52.70 (12.02–231.1)	69.57 (8.32–581.9)	.95	0.52 (.19–1.42)	0.9 (.2–4.03)	.66
6B	39.97 (7.27–219.6)	65.58 (5.72–752.1)	.76	0.39 (.2–.76)	0.55 (.17–1.83)	.75
14	169 (37.31–765.2)	223 (32.02–1553)	.82	0.46 (.2–1.08)	0.84 (.21–3.3)	.48
23F	48.82 (10.19–233.9)	78.70 (7.61–813.5)	.80	0.38 (.18–.79)	0.51 (.15–1.77)	.99
6A	46.37 (5.9–364.3)	74.07 (5.68–966.9)	.72	0.29 (.16–.52)	0.51 (.15–1.8)	.32
9V	65.79 (11.18–387.1)	82.85 (9.02–761.2)	.93	0.4 (.17–.91)	0.74 (.19–2.9)	.42
18C	55.35 (11.88 – 258)	70.12 (8.6–570.9)	.80	0.71 (.24–2.07)	1.17 (.18–7.48)	.99
19F	74.04 (14.81–370.2)	92.55 (11.48–746)	.93	0.65 (.3–1.42)	1.08 (.31–3.81)	.49
1	11.22 (6.65–18.92)	16.39 (6.35–42.26)	.49	2.19 (.96–5.02)	2.5 (.65–9.74)	.82
5	17.79 (6.98–45.31)	26.98 (7.97–91.28)	.44	0.53 (.23–1.21)	0.62 (.136–2.84)	.83
7F	142.4 (25.17–805.4)	117.4 (10.02–1376)	.87	0.63 (.21–1.86)	1.20 (.19–7.50)	.66
19A	23.52 (6.64–83.31)	47.49 (5.76–391.3)	.63	1.11 (.33–3.72)	2.31 (.4–13.37)	.50
3	22.37 (8.4–59.54)	24.31 (6.58–89.82)	.90	0.32 (.15–.7)	0.56 (2–1.63)	.48

Abbreviations: CI, confidence interval; GMC, geometric mean concentration; GMOI, geometric mean opsonophagocytic index; HEU, human immunodeficiency virus-exposed uninfected; HU, human immunodeficiency virus-unexposed uninfected; PCV13, pneumococcal conjugate vaccine 13.

a The GMOI and 95% CIs of results are reported for MOPA (HU n = 11, HEU n = 9); the serotype-specific IgG GMC (HU, n = 11; HEU, n = 8) for all PCV13 serotypes (1, 3, 4, 5, 6 A, 6B, 7F, 9V, 14, 18C, 19A, 19F, and 23F) are reported, and less than 8 was reported as no response.

**Figure 5.**
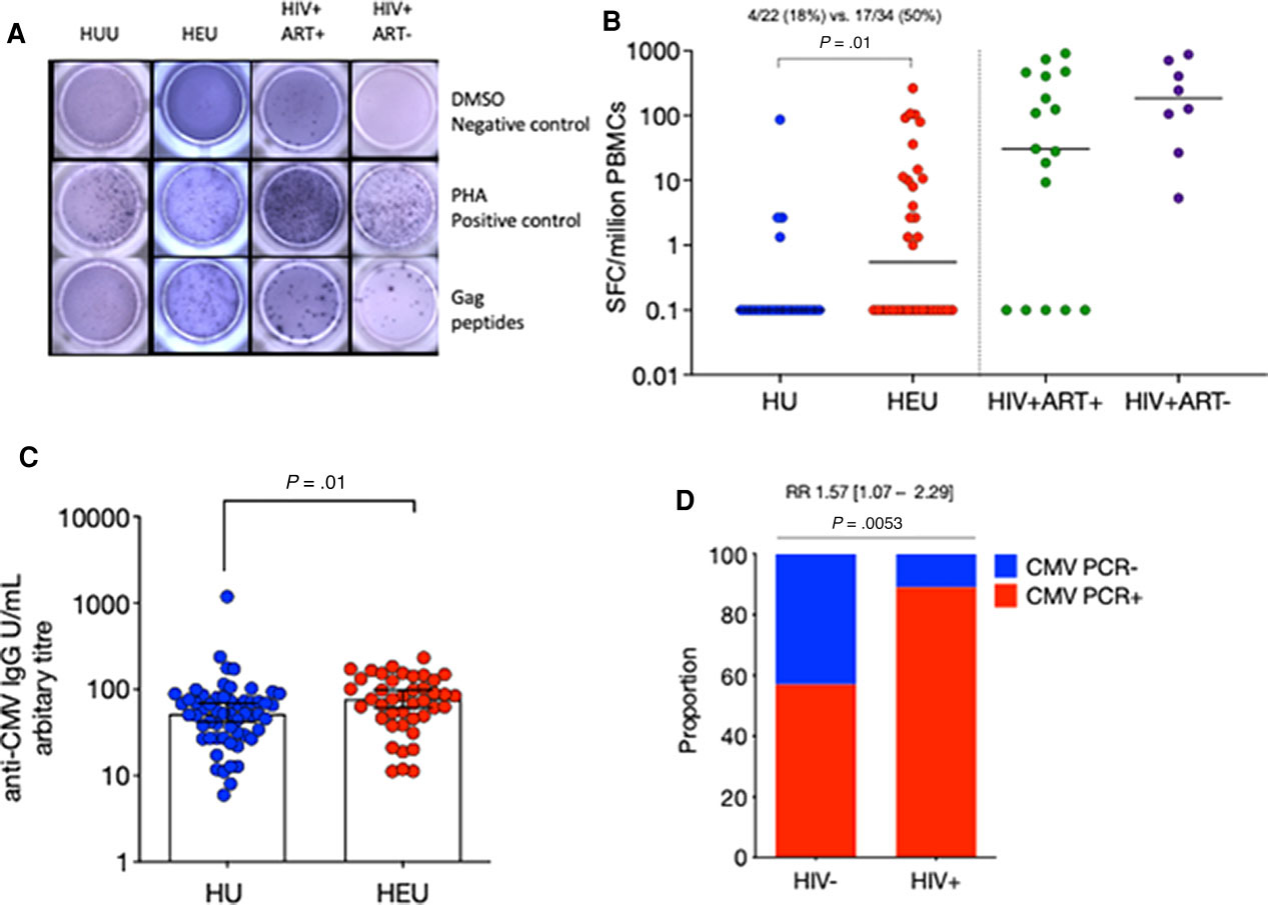
Infant exposure to immune-modulating viruses human cytomegalovirus (CMV) and human immunodeficiency virus (HIV). (*A*) Detection of Gag-specific T cells by T-cell enzyme-linked immunospot (ELISpot) assay in human immunodeficiency virus-unexposed uninfected (HU) and human immunodeficiency virus-exposed uninfected (HEU) infants and antiretroviral therapy (ART)-naive/experienced HIV-infected adults. Isolated peripheral blood mononuclear cells (PBMCs) were incubated with 15-mer Gag peptide pool, phytohemagglutinin (PHA) as a positive control, or Roswell Park Memorial Institute media as a negative control. Interferon-γ-producing cells were detected on a 96-well microtiter ELISpot plate. (*B*) The frequency of spot-forming cells (SFCs)/million PBMCs are plotted for all subjects. Data were analyzed using Fisher’s exact test (HU, n = 22; HEU, n = 34; HIV^+^ART^+^, n = 17; HIV^+^ART^−^, n = 8). (*C*) Plasma anti-human cytomegalovirus (CMV) immunoglobulin G (IgG) was measured in infants between 5 and 15 weeks of age and assigned an arbitrary titer. The proportion of (*D*) HIV-infected and uninfected mothers with reverse-transcription polymerase chain reaction (RT-PCR) detected CMV deoxyribonucleic acid in their breast milk, (HIV^−^, n = 23; HIV^+^, n = 35). The IgG data were analyzed using Mann-Whitney *U* test reported as medians (interquartile range) and PCR data using a Fisher’s exact test reporting effective size as a relative risk (RR). Blue circles are HU controls and red circles are HEU. Data are presented as means (standard deviation). DMSO, dimethyl sulfoxide.

To determine potential drivers of the described immune alterations in newborns and infants, we measured HIV Gag-specific responses in cord and peripheral blood mononuclear cells [29] using an 18-hour ex vivo IFNγ ELISpot assay in both the HEU newborn and the longitudinal infant cohorts (Figure 5A and B; Supplementary Figure 7A) (HU, n = 22; HEU, n = 34; HIV^+^ART^+^, n = 17; HIV^+^ART^−^, n = 8) and (HU, n = 35; HEU, n = 23), respectively. Few Gag-specific responses were detectable in cord blood of HEU newborns (*P* = .05) (Supplementary Figure 7A). However, in older infants, there was detectable IFNγ producing HIV Gag-specific cell responses in approximately 50% of the longitudinal cohort of HEU infants (mean 22 [50%] vs 4.3 [18%]; *P* = .01) (Figure 5B).

We then assessed the association of EBV/CMV exposure as a potential driver of immune alterations. We found that anti-hCMV IgG titers were higher in plasma from HEU infants aged 5–15 weeks than HU controls (86% vs 79.8%, *P* = .012) (Figure 5C) (HU, n = 57; HEU, n = 42); however, hCMV PCR detection in throat swabs was similar using a χ^2^ test (29% vs 14%, *P* = .19) (Supplementary Figure 8A) (HU, n = 19; HEU n = 33), indicating potential differences in the pattern of exposure to hCMV. In maternal breastmilk, we found a higher proportion of hCMV PCR-positive HIV-infected mothers compared with HIV-uninfected controls (31 [89%] vs 13 [57%]; *P* = .005) (Figure 5D) (HIV^−^, n = 57; HIV^+^, n = 42).

Epstein-Barr virus seropositivity is rapid up to 1 year of life [21]. In our infant cohort, EBV VCA detection by anti-IgG (*P* = .07) was high in plasma but PCR detection was low (*P* = .22) in throat swabs; EBV results were not significantly different between HEU and HU groups (Supplementary Figure 8B and C) (HU, n = 24; HEU n = 34). Taken together, HHV (hCMV and EBV) infection are common in infancy in this setting, irrespective of HIV-exposure status.

## DISCUSSION

Human immunodeficiency virus-exposed but uninfected infants are at an increased risk of infectious disease even in the era of universal access to maternal ART; however, the underlying immunological basis is not well understood. We show altered monocyte phagosomal function, dysregulated B-cell homeostasis, and selective impairment of vaccine responses in HEU infants within the first 6 months of life. We also demonstrate evidence of HIV exposure and increased likelihood of hCMV exposure in HEU infants. We postulate that the variable severity and/or persistence of this immunological phenotype may explain the variable clinical manifestations reported in HEU infants [31, 32], which may depend on the duration and intensity of exposure to HIV and other infectious cofactors.

The impaired monocyte phagosomal function in HEU newborns highlights their potential vulnerability to bacterial infection before the primary immunization series. Monocyte bactericidal activity requires uptake, reactive oxygen species formation, and phagosomes-lysosomes fusion resulting in inhibition, killing, and degradation of internalized bacteria [33]. In our setting, the validated flow cytometer reporter assay of phagocyte function that uses zymosan has shown poor immune function and superoxide burst activity in HIV-infected adults with active TB [34]. Monocyte functional impairment against encapsulated bacteria has also been observed in “age-associated” inflammation, where monocyte-activating cytokines tumor necrosis factor (TNF)-α and interleukin (IL)-6 are augmented [35]. Likewise, increased monocyte inflammatory markers, including sTNF-RI, IL-6, IP-10, oxLDL, and sCD14 are reported in HEU newborns [33], as well as enhanced proinflammatory cytokine secretion after stimulation with diverse pathogen-associated molecular pattern molecules at 6 weeks of age [36]. Moreover, recent PBMC transcriptomic profiling in HEU infants aged 1–2 years revealed down-regulated genes (LCN2, CAMP, HP, MMP8, BPI, LTF) associated with neutrophil function [37]. Taken together, this may explain HEU infant increased susceptibility to bacterial infection and pneumonia.

We also found increased FcLR4 expression on B cells. In chronically HIV-infected adults, the inhibitory BCR FcLR4 are overrepresented, an “exhausted” B-cell phenotype, with poor BCR-mediated activation and antigen-specific antibody production [38, 39]. In HEU infants, at 6 to 14 weeks of age, we observed low proportions of tissue-like memory and immature-transitional B-cell subsets, which, conversely, are augmented in chronically HIV-infected adults [40, 41] and are indicative of dysregulated B-cell homeostasis. More importantly, our data present evidence of increased exposure to HIV and hCMV in HEU infants from maternal HIV and/or viral proteins, hCMV recrudescence, and high PPD-specific IFNγ, which may promote B-cell dysregulation in early life. The PPD responses are shown to be increased in *Mycobacterium tuberculosis*-sensitized mothers, [42] and a bimodal response to BCG/PPD (high/low) has been reported in HEU infants in our setting [43]; BCG vaccination induces heterologous effects in myeloid cells at an epigenetic level in a process termed “trained immunity” [44], which may explain our findings. Taken together, the mechanisms of B-cell dysregulation are likely distinct from those seen in chronic HIV infection (hypergammaglobulinemia), due to the lack of replicative virus and preserved CD4 T cells.

Altered B-cell homeostasis is associated with impaired antibody responses during chronic HIV infection [45, 46]. Consistent with this observation, HIV-infected mothers in our cohort exhibited lower anti-Hib and anti-TT antibody titers than HIV-uninfected mothers using DPT-HepB-Hib. In agreement with maternal titers, we observed low anti-Hib titers in HEU infants. However, our observation is in contrast with studies conducted in South Africa [47] and Uganda [48, 49], who reported robust anti-Hib and anti-DT antibody titers in HEU infants. Differential vaccine immunogenicity is likely multifactorial, influenced by persistent immune exposure to HIV proteins, the time to maternal ART use, and the unique burden of infectious cofactors that likely contribute to a microenvironment of proinflammation. Consistent with previous observations [50], we also observed that a relatively large number of HEU infants mounted an IFNγ response after stimulation with HIV Gag; however, we did not detect IFNγ responses in HEU newborns, and responses were poor to the T-cell mitogen PHA (which cross-links the TCR/glycosylated surface proteins). Taken together, these data point towards HIV exposure as a possible driver of selective T-cell regulation at birth.

Our study limitations include the following: the limited number of assays per sample restricted adjustment for multiple comparisons; we excluded premature/low-birth weight/small for gestational age babies; no maternal viral load or clinical presentation of disease were taken; and HIV-negative mothers were not retested at the study end.

## CONCLUSIONS

In conclusion, we show altered monocyte phagosomal function, dysregulated B-cell homeostasis, and selective impairment of vaccine antibody responses in HEU infants within the first 6 months of life. This period of vulnerability likely contributes to increased susceptibility to disease-causing bacteria that commonly cause life-threating illness such as pneumonia in HEU infants.

## Supplementary Data

Supplementary materials are available at *The Journal of Infectious Diseases* online (http://jid.oxfordjournals.org/). Supplementary materials consist of data provided by the author that are published to benefit the reader. The posted materials are not copyedited. The contents of all supplementary data are the sole responsibility of the authors. Questions or messages regarding errors should be addressed to the author.

## Supplementary Material

jiac133_Supplementary_Data

## Notes

***Acknowledgments.*** We are grateful to the participants for their willingness to participate in this study.

***Author contributions.*** L. A., A. F., R. S. H., S. R.-J., and D. J. C. M. contributed to study design. L. A., A. P., B. F., R. K., D. H. B., D. G., P. M., and D. J. C. M. performed the experiments. W. N. recruited and monitored the participants. L. A. and K. C. J. contributed to analysis and interpretation. H. C. M., D. J. C. M., A. F., K. C. J., and R. S. H. reviewed the manuscript.

***Disclaimer.*** The views expressed are those of the author(s) and not necessarily those of the National Institute for Health and Care Research (NIHR) or the UK Government Department of Health and Social Care.

***Financial support.*** This work was funded by the Wellcome Trust and a studentship from the Franklin Adams Trust. The Malawi-Liverpool-Wellcome Trust Clinical Research Programme was supported by a strategic award from the Wellcome Trust. R. S. H. is an NIHR Senior Investigator. Funding to pay the Open Access publication charges for this article was provided by the Jisc Read and Publish deal.
