# Supplementary material for: Defective Monocyte Enzymatic Function and an Inhibitory Immune Phenotype in Human Immunodeficiency Virus-Exposed Uninfected African Infants in the Era of Antiretroviral Therapy
Source: J Infect Dis. 2022 Apr 11;226(7):1243–55. doi: 10.1093/infdis/jiac133 (PMC9518837; doi:10.1093/infdis/jiac133)
Supplement: jiac133_Supplementary_Data [file jiac133_supplementary_data.zip › Supplementary_materials_clean210322.docx]

**Supplementary materials**

**Defective monocyte enzymatic function and an inhibitory immune phenotype in HIV-exposed uninfected African infants in the era of antiretroviral therapy**

Louise Afran^1,2,3^, Kondwani C. Jambo^1,3^, Wilfred Nedi^1^, David JC Miles^1,4^, Anmol Kiran^1, 7^, Dominic H Banda^1^, Ralph Kamg’ona^1^, Dumizulu Tembo^1^, Annette Pachnio^4^, Eleni Nastouli^6^, Brigit Ferne^6^, Henry C Mwandumba^1,3^, Paul Moss^4^, David Goldblatt^6^, Sarah Rowland-Jones^5^, Adam Finn^1^, Robert S Heyderman^1, 6^

Running title: Immune dysregulation in HEU African infants

Summary: Early life HIV-exposure may dysregulate innate and adaptive immunity, specifically, monocyte function and vaccine induced immunity to encapsulated bacteria. This may lead to altered protection and susceptibility to disease from encapsulated bacteria.

**Supplementary methods**

**1a. Measurement of monocyte phagosomal enzymatic activity**. Phagosomal oxidative burst and bulk proteolytic function in monocytes was measured using a flow cytometry–based reporter bead assay as described previously [1–3]. 3μm diameter silica beads were derivatized with the calibration fluorochrome (Alexa Fluor 405-SE) and the fluorogenic reporter substrates Oxyburst Green succinimidyl ester (Oxyburst-SE) (Molecular Probes, Eugene, OR) for superoxide burst, or DQ Green bovine serum albumin (DQ-beads) (Molecular Probes, Eugene, OR) for bulk proteolysis. For each assay, 10ul of Oxyburst-SE (superoxide burst) or DQ bovine serum albumin (bulk proteolysis) beads suspension were added to 100ul of cord blood. When the beads are internalised by monocytes the oxidation-sensitive fluorescent reporter Oxyburst-SE fluoresces and the fluorescence intensity is proportional to the degree of activity in the phagosome, which provided a readout of phagosomal enzymatic activity. For proteolysis the DQ green is unquenched and the fluorescence proportional to bulk proteolysis. Cells were collected at two time points for superoxide burst 10 and 60 minutes or bulk proteolysis 10 and 240 minutes. The readout for the assay is reported as the ratio of the median fluorescent intensity of the reporter fluorochrome at 60mins:10mins for oxidative burst and 240mins:10mins for bulk proteolysis. The uptake cut-off was 35% in the assay. Data are reported as median[95%CI].

**1b. T cell IFNγ ELISpot**. 2x10^6^ PBMCs were stimulated in an 18-hour IFN-γ T cell ELISpot as previously described [4]. The enumerated IFN-γ producing cells after antigen stimulation were used as an index of effector memory T cell responses. Data were reported after deducting 2x the value of the negative well. Stimulating antigens were: Phaseolus vulgaris lectin (PHA) 5ug/ml (NIBSC, UK), purified protein derivative (PPD) 10ug/ml (Statens serum institute, Denmark) and HIV-1 consensus C gag 15-mer peptides (GAG peptide) 10ug/ml (NIH AIDS, USA)[4]

**1c. Human cytomegalovirus PCR** . Real time PCR was used to detect hCMV in HIV-infected and uninfected maternal breast milk and infant oropharangeal swabs.

The results were based on a standard curve constructed using an in-house plasmid previously calibrated against quality controls for molecular diagnostics and external quality assessment panels ([5]). The positive control was assessed again and an expected value of 2,500 copies seen with results read on the ABI Prism 7500 (Thermofisher, UK). The amplification plot was assessed against a standard slope range of -3.10 to -3.60 and a R^2^ value > 0.9.

**1d.** **Detection of Cytomegalovirus specific IgG and IgM antibodies**. A semi-quantitative, in-house hCMV IgG assay was used at the laboratories in the University of Birmingham, UKPlasma was diluted 1/4 in a dilution in a 1:1:1 mix of plasma from three healthy donors, the top concentration was assigned an arbitrary unit of 1000. The unknown samples were related to the standard curve and a titre calculated. Briefly, Nunc 96-well plates Maxisorb (Fisher Scientific # 442404) were coated with 50μL UV activated hCMV-lysate (1:4000) and mock-lysate (1:4000) in carbonate-bicarbonate buffer pH 9.6 (sigma capsules), then covered with parafilm and incubated at 4^o^C overnight in the fridge. The following day, plates were washed three times in 200μL (PBS + 0.05% Tween20), 100μL of samples were added 1:600 in dilution buffer (PBS + 1%BSA + 0.05% Tween20), blank dilution buffer, and standards, then incubated for 1h at room temperature (RT). Plates were washed again three times, then 100μL of the secondary antibody, anti-human IgG-HRP (1/8000 dilution in PBS+1% BSA+0.05% Tween20) goat anti- human IgG (Southern Biotech #2040-05). Plates were incubated and washed again as described after which 100μL of tetramethyl benzidine (TMB-solution) (Tebu-Bio) was added for 10 min at RT. The reaction was stopped with 100μL 1M HCl and plates were read at 405nm (Biotek instrument).

**1e. Sandwich enzyme-linked immunosorbent assay to detect IgG specific to vaccine antigens**. In an in-house ELISA, TT or DT (both NIBSC, UK) were diluted to 0.5 Lf/mL or Hib capsular polysaccharide 0.1μg/mL in 10 mL carbonate coating buffer (0.015 M Na_2_CO_3_, 0.035 M NaHCO_3_ pH9.6); 100 μL per well of the solution was pipetted into a 96-well flat bottom Maxisorp plate. Plates were incubated overnight at 4 ̊C then washed seven times with PBS 0.05% Tween. 50 μl goat anti-human alkaline phosphatase-conjugated secondary antibodies (Southern Biotech, UK) were diluted to 1ug/mL in PBS 0.05% Tween 2% BSA and added to each well. The plates were incubated for 1 h at 37 ̊C, then washed seven times with PBS 0.05% Tween. 100 μL of Sigma-fast p-nitrophenyl phosphate substrate was added to each well (Sigma, UK). A standard curve was generated using a set of 2- fold dilutions of a standard pooled serum (NIBSC, UK). Optical density was measured (without acid stopping the reaction) after 10 min using an ELISA plate reader (Biotek, UK) set at 405nm and SoftMax Pro software [6]. Minimum putative protective titres are 0.15ug/mL (passive) and 1.0ug/mL (acquired) for *Hib* [7], and 0.01IU/mL for TT[8] and DT[9]. Median[95%CI) reported.

**1f. Multiplexed opsonophagocytosis killing assay and serotype-specific IgG** . Sera from 20 infants were sent on dry ice to the UCL Great Ormond Street Institute of Child Health (UCL; United Kingdom). Infants had received 3 doses of Prevnar (PCV13) at 6, 10, and 14 weeks of age. Sera were stored at –20°C until analysis. Analyses were performed at the World Health Organization pneumococcal reference laboratory (University College London, United Kingdom). Immunoglobulin G (IgG) serum concentrations specific for the 13 vaccine serotypes (1, 3, 4, 5, 6 A, 6B, 7F, 9V, 14, 18C, 19A, 19F, and 23F) were measured using an enzyme-linked immunosorbent assay (ELISA) after adsorption with cell-wall and 22F polysaccharides to increase the assay specificity [10]. A standardized opsonophagocytic assay (OPA) was used to measure functional antibodies against the same serotypes [11], described in supplementary methods 1d. The OPA titre was defined as the reciprocal of the lowest serum dilution that induces ≥50% bacterial cell death compared to the assay control.

**Supplementary Tables**

**Supplementary Table 1. Fluorochromes**

**Supplementary Table 2. Fluorescent antibody panels**

**Supplementary Figures**

**Supplementary Figure 1: Gating strategy to detect monocyte phagosomal functional capacity at birth.** Singlets were defined using FSC-A vs. FSC-H parameters and lymphocytes were gated using SSC-A and CD14 PE-CY7. Then FSC-A vs. AF405 to gate cells with beads. The readout for the assay are reported as the median fluorescent intensity of the reporter fluorochrome at 60mins:10mins and 240mins:10mins for oxidative burst and bulk proteolysis, respectively. The activity index was calculated using a ratio of the reporter fluorochrome over the the calibration fluorochrome. Only individuals with an uptake of greater than ≥30% were used in the phagosomal analysis. Data are reported as median [95%CI].

**Supplementary figure 2: Characterisation of B cell and T cell subsets in HEU and HU infants**

Using peripheral blood collected from the longitudinal infant cohort aged 5-9 weeks, whole blood was stained with the following fluorochrome-conjugated antibodies, anti-CD19 APC, anti-CD10 PE-Cy7, anti-CD21-FITC and anti-CD27 PE. Singlets were defined using FSC-A vs. FSC-H parameters and lymphocytes were gated using SSC-A and FSC-A. B cells were then gated using CD19 against SSC-A. **a)** The proportion of B cell subsets were clasified using CD10, CD21 and CD27 as follows**:** CD10^-^CD21^+^CD27^-^ (naive), CD10^-^CD21^-^CD27^+^ (resting memory), CD10^-^CD21^+^CD27^+^ (activated memory), CD10^-^CD21^-^CD27^-^ (tissue-like memory) and **c)** CD10^+^CD27^-^ (immature transitional).

The proportion of T cells were measured with the following fluorochrome-conjugated antibodies, anti-CD3 APCH7, anti-CD4 Pacific Blue, anti-CD8-FITC, anti-CCR7 APC and anti-CD45RA PE. Singlets were defined using FSC-A vs. FSC-H parameters and lymphocytes were gated using SSC-A and FSC-A. T cells were then gated using CD3 against SSC-A, then a CD4 versus CD8 plot was used to separate the two main T cell subsets. **b)**CD4^+^ and **c**) CD8^+^ T cell subsets were clasified using CCR7 and CD45RA as follows CCR7-CD45RA- (effector memory), CCR7^+^CD45RA- (central memory), CCR7^+^CD45RA^+^ (naïve) and CCR7^-^CD45RA^+^(terminally-differentiated). Data are presented as medians [IQR] and analysed using Mann Whitney U test (HU n=42, HEU n=31).

**d**) IFN-γ producing cells were measured using a T-cell ELISPOT assay in HU and HEU infants. Isolated PBMCs were incubated with either PPD, TT, Hb or PHA as a positive control or RPMI media as a negative control for 18 hours. IFN-γ producing cells were detected on a 96-well microtitre ELISpot plate. The frequency of SFCs/million PBMCs are plotted for all subjects. Data analysed using Fisher’s exact test (HU, n =22; HEU, n=34), median[95%CI) reported.

**Supplementary Figure 3: Gating strategy to characterise B cells from infant blood. a)** Blood was stained with the following fluorochrome-conjugated antibodies, anti-CD19 APC, anti-CD10 PE-Cy7, anti-CD21-FITC and anti-CD27 APC-CY7. Singlets were defined using FSC-A vs. FSC-H parameters and lymphocytes were gated using SSC-A and FSC-A. B cells were then gated using CD19 against SSC-A. The following populations were determined: CD10^-^CD21^+^CD27^-^ (naive), CD10^-^CD21^-^CD27^+^ (resting memory), CD10^-^CD21^+^CD27^+^ (activated memory), CD10^-^CD21^-^CD27^-^ (tissue-like memory) and CD10^+^CD27^-^ (immature transitional). **b)** Blood was stained with CD19 PERCP, anti-CD10 PE-Cy7, anti-CD21-FITC and anti-CD27 APCCY7, FcRL4 PE, CD95e450 and CD27 APCCY7 to determine B cells expressing inhibitory markers. **M**edian[95%CI) reported.

**Supplementary Figure 4: Gating strategy to characterise B cells from cord blood. a)** Blood was stained with the following fluorochrome-conjugated antibodies, anti-CD19 APC, anti-CD10 PE-Cy7, anti-CD21-FITC and anti-CD27 PE. Singlets were defined using FSC-A vs. FSC-H parameters and lymphocytes were gated using SSC-A and FSC-A. B cells were then gated using CD19 against SSC-A. The following populations were determined: CD10^-^CD21^+^CD27^-^ (naive), CD10^-^CD21^-^CD27^+^ (resting memory), CD10^-^CD21^+^CD27^+^ (activated memory), CD10^-^CD21^-^CD27^-^ (tissue-like memory) and CD10^+^CD27^-^ (immature transitional). **b)** Blood was stained with CD19 PERCP, anti-CD10 PE-Cy7, anti-CD21-FITC and anti-CD27 APCCY7, FcRL4 PE, CD95e450 and CD27 APCCY7 to determine B cells expressing inhibitory markers. Median[95%CI) reported.

**Supplementary Figure 5: Gating strategy to characterise T cell subsets. a)**Whole blood was stained with the following fluorochrome-conjugated antibodies, anti-CD3 APCH7, anti-CD4 Pacific Blue, anti-CD8-FITC, anti-CCR7 APC and anti-CD45RA PE. **b)** Cord blood was stained with the following fluorochrome-conjugated antibodies, anti-CD3 APCY7, anti-CD4 Pacific Blue, anti-CD8-PECY7, anti-CCR7 APC and anti-CD45RA PECY5, PD-1 PE and CD57 FITC.

Singlets were defined using FSC-A vs. FSC-H parameters and lymphocytes were gated using SSC-A and FSC-A. T cells were then gated using CD3 against SSC-A, then a CD4 versus CD8 plot was used to separate the two main T cell subsets. FMO were used to determine gating. CD4^+^ and CD8^+^ T cell subsets were clasified using CCR7 and CD45RA as follows CCR7-CD45RA- (effector memory), CCR7^+^CD45RA- (central memory), CCR7^+^CD45RA^+^ (naïve) and CCR7^-^CD45RA^+^(terminally-differentiated). Median[95%CI) reported.

**Supplementary Figure 6: Infant and maternal antibody responses.** Preceding and following Penta-DTwPHibHepB vaccination we measured vaccine titers using an ELISA to **a**) anti-TT IgG and **b**) anti-DT IgG in infants at 5-7 (HU, n=50; HEU n=39), 14-15 (HU, n=22; HEU, n=27) and 18-23 (HU, n=25; HEU, n=19) weeks. HIV- uninfected (n=61) and HIV-infected (n=43) maternal titres were measured for **c**) anti-DT IgG when infants were aged 5-7 weeks old. Blue circles are controls and red are HEU infants or HIV-infected mothers. Green dotted horizontal line represents cut-off for protective titers. Percentage of seropositivity is depicted inbar charts. Data are presented as medians and analysed using Mann Whitney U test. Minimum putative protective titres are 0.15ug/mL (passive) and 1.0ug/mL (acquired) for *Hib* [50], and 0.01IU/mL for TT and DT. Median[95%CI) reported.

**Supplementary Figure 7: IFN-γ producing cells to HIV protein GAG at birth**

A 15-mer GAG peptide pool was used to stimulate isolated cord blood mononuclear cells from cord blood in an 18hour T cell EliSpot assay. IFN-γ producing cells were detected on a 96-well microtitre ELISpot plate. **a)** The frequency of GAG SFCs/million cord blood mononuclear cells are plotted for all subjects. Data analysed using Fisher’s exact test (HU, n =35; HEU, n=23). Median[95%CI) reported.

**Supplementary Figure 8: Infant exposure to immune modulating viruses HCMV and EBV.** HCMV and EBV viral DNA was measured by RT-PCR and anti-EBV IgG using an ELISA: **a)** Infant oropharangeal throat swab CMV DNA RT-PCR, (HU, n =19; HEU n=33), **b)** infant serum anti-EBV IgG and **c**) HIV-infected and uninfected mothers with EBV RT-PCR detected in breast milk, (HIV- n=24, HIV+ n=34). Data analysed using Fisher’s exact test and effective size reported as a relative risk. Median[95%CI) reported.

**References**

1. Jambo KC, Banda DH, Kankwatira AM, et al. Small alveolar macrophages are infected preferentially by HIV and exhibit impaired phagocytic function. Mucosal Immunol [Internet]. **2014** [cited 2014 Mar 20]; . Available from: http://www.ncbi.nlm.nih.gov/pubmed/24472847

2. Yates RM, Hermetter A, Russell DG. The kinetics of phagosome maturation as a function of phagosome/lysosome fusion and acquisition of hydrolytic activity. Traffic [Internet]. Traffic; **2005** [cited 2020 Jul 18]; 6(5):413–420. Available from: https://pubmed.ncbi.nlm.nih.gov/15813751/

3. Podinovskaia M, Vanderven BC, Yates RM, et al. Dynamic quantitative assays of phagosomal function. Curr Protoc Immunol [Internet]. **2013** [cited 2014 Mar 20]; 102:14.34.1-14.34.14. Available from: http://www.ncbi.nlm.nih.gov/pubmed/24510516

4. Kabilan L, Andersson G, Lolli F, Ekre H, Olsson T, Troye-Blomberg M. Detection of intracellular expression and secretion of interferon-gamma at the single-cell level after activation of human T cells with tetanus toxoid in vitro. Eur J Immunol. **1990**; 20(5):1085–9.

5. Hamprecht K, Vochem M, Baumeister A, Boniek M, Speer CP, Jahn G. Detection of cytomegaloviral DNA in human milk cells and cell free milk whey by nested PCR. J Virol Methods [Internet]. **1998** [cited 2014 Apr 25]; 70(2):167–76. Available from: http://www.ncbi.nlm.nih.gov/pubmed/9562410

6. Engvall E, Perlmann P. Enzyme-linked immunosorbent assay (ELISA). Quantitative assay of immunoglobulin G. Immunochemistry [Internet]. **1971** [cited 2018 Dec 17]; 8(9):871–4. Available from: http://www.ncbi.nlm.nih.gov/pubmed/5135623

7. WHO. Weekly epidemiological record Relevé épidémiologique hebdomadaire [Internet]. 2006. Available from: http://www.who.int/wer

8. WHO. Tetanus vaccines: WHO position paper – February 2017. **2017** [cited 2020 Sep 16]; 92(6):53–76. Available from: http://www.who.int/immunization/sage/meet-ings/2016/october/presentations_background_docs/en/

9. WHO. Diphtheria vaccine: WHO position paper – August 2017. **2017** [cited 2020 Sep 16]; 92(31):417–436. Available from: https://apps.who.int/iris/bitstream/handle/10665/258681/WER9231.pdf?sequence=1

10. Wernette CM, Frasch CE, Madore D, et al. Enzyme-linked immunosorbent assay for quantitation of human antibodies to pneumococcal polysaccharides. Clin Diagn Lab Immunol [Internet]. American Society for Microbiology; **2003** [cited 2019 Feb 8]; 10(4):514–9. Available from: http://www.ncbi.nlm.nih.gov/pubmed/12853378

11. Rose CE, Romero-Steiner S, Burton RL, et al. Multilaboratory comparison of Streptococcus pneumoniae opsonophagocytic killing assays and their level of agreement for the determination of functional antibody activity in human reference sera. Clin Vaccine Immunol [Internet]. American Society for Microbiology; **2011** [cited 2019 Feb 8]; 18(1):135–42. Available from: http://www.ncbi.nlm.nih.gov/pubmed/21084458
