# Supplementary figures and images for: Defective Monocyte Enzymatic Function and an Inhibitory Immune Phenotype in Human Immunodeficiency Virus-Exposed Uninfected African Infants in the Era of Antiretroviral Therapy
Source: J Infect Dis. 2022 Apr 11;226(7):1243–55. doi: 10.1093/infdis/jiac133 (PMC9518837; doi:10.1093/infdis/jiac133)

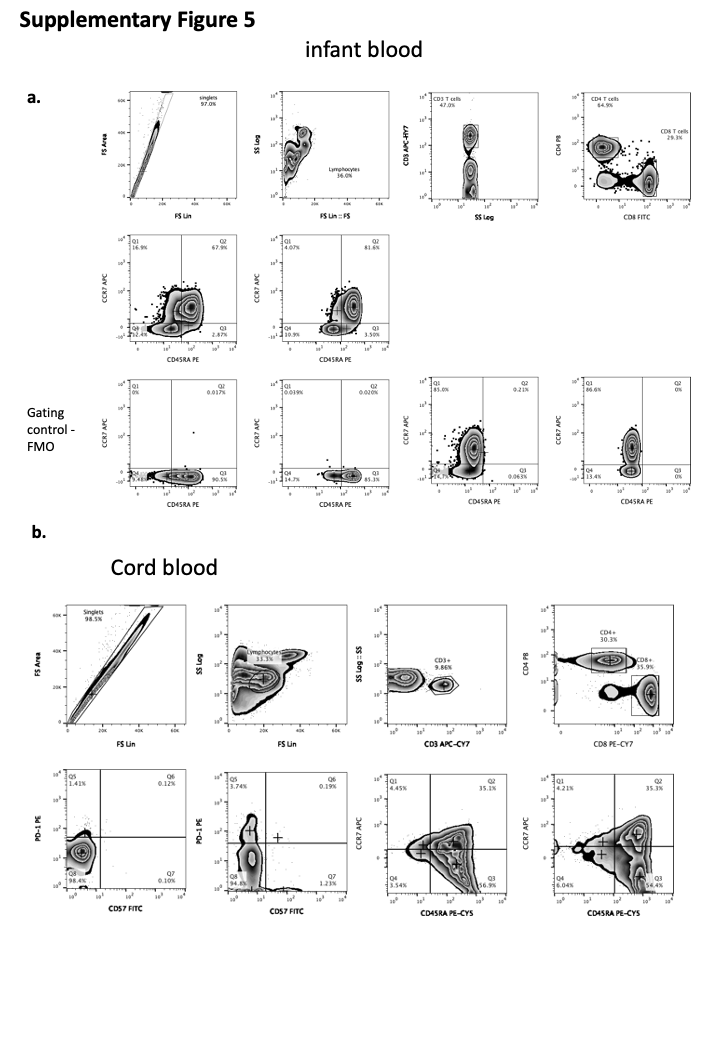

Supplement: jiac133_Supplementary_Data [file jiac133_supplementary_data.zip › Slide10.tiff]

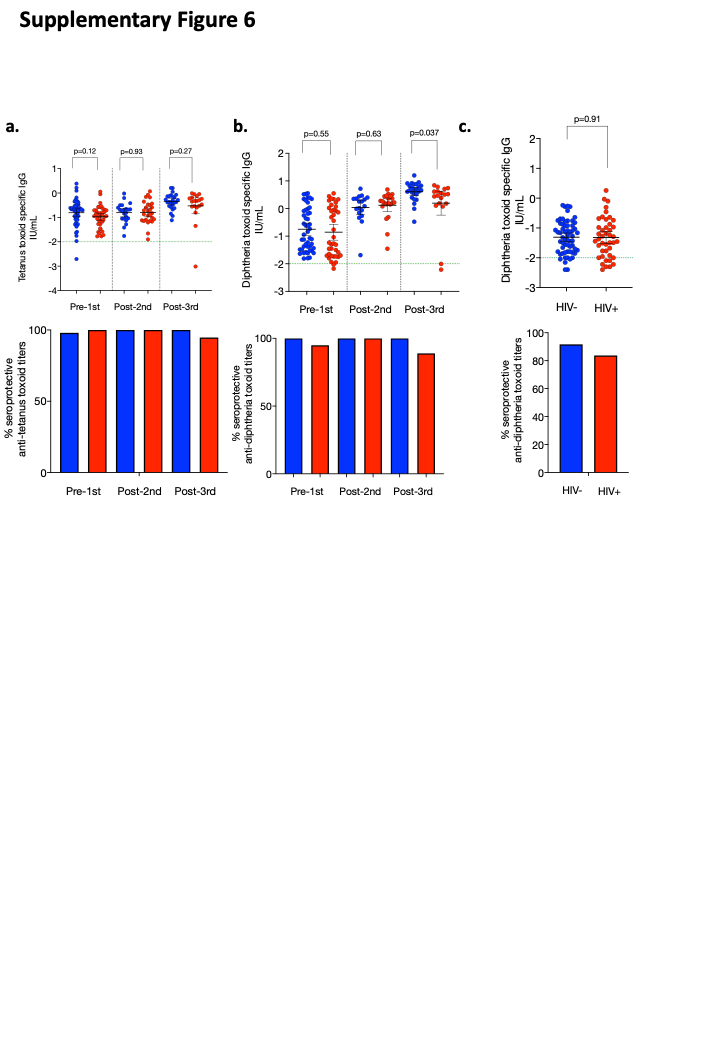

Supplement: jiac133_Supplementary_Data [file jiac133_supplementary_data.zip › Slide11.tiff]

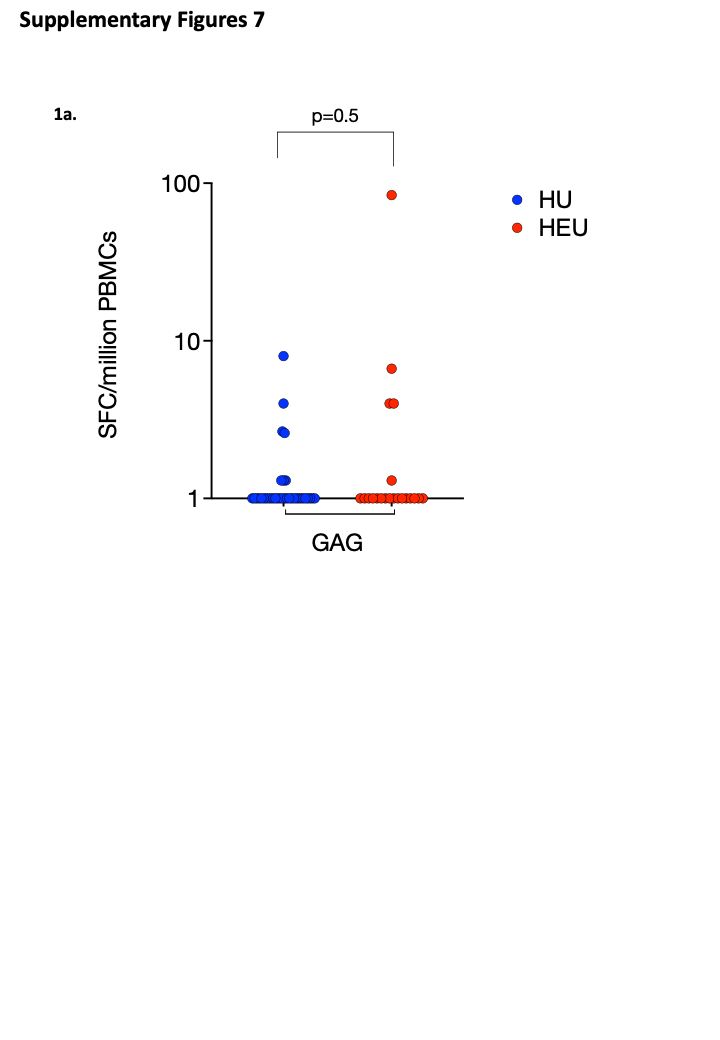

Supplement: jiac133_Supplementary_Data [file jiac133_supplementary_data.zip › Slide12.tiff]

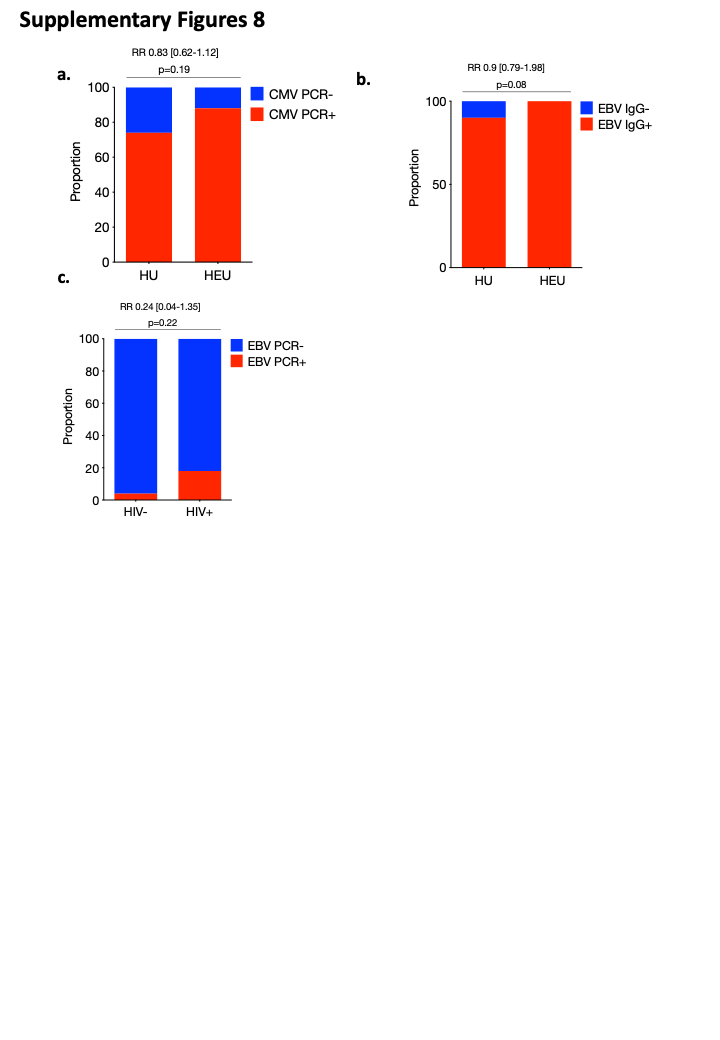

Supplement: jiac133_Supplementary_Data [file jiac133_supplementary_data.zip › Slide13.tiff]

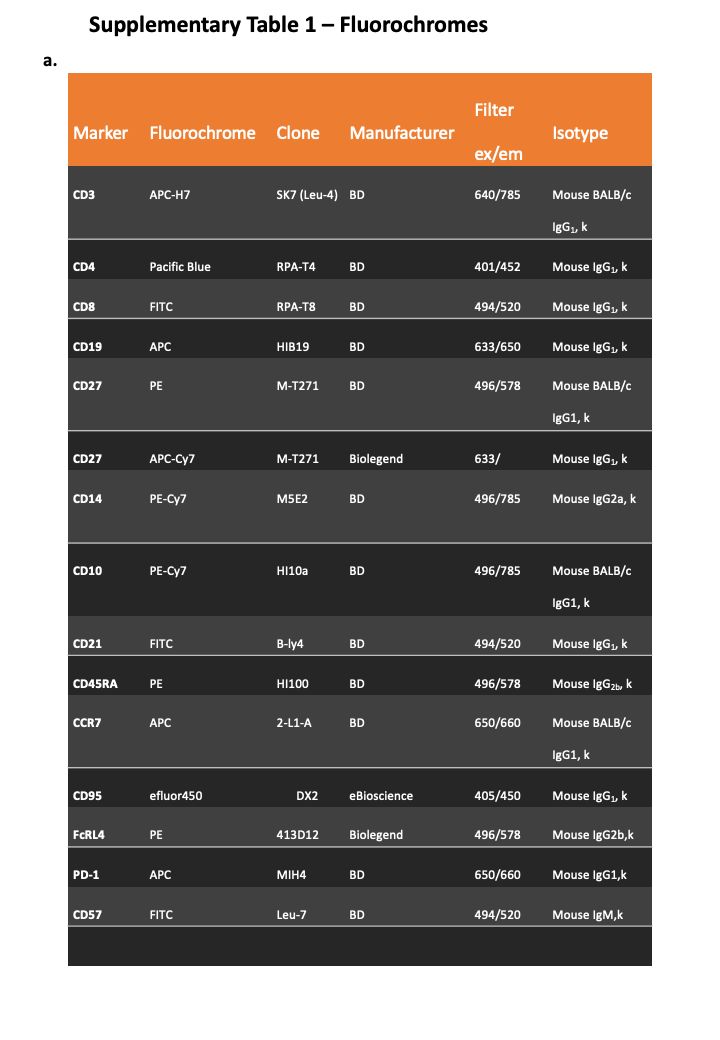

Supplement: jiac133_Supplementary_Data [file jiac133_supplementary_data.zip › Slide14.tiff]

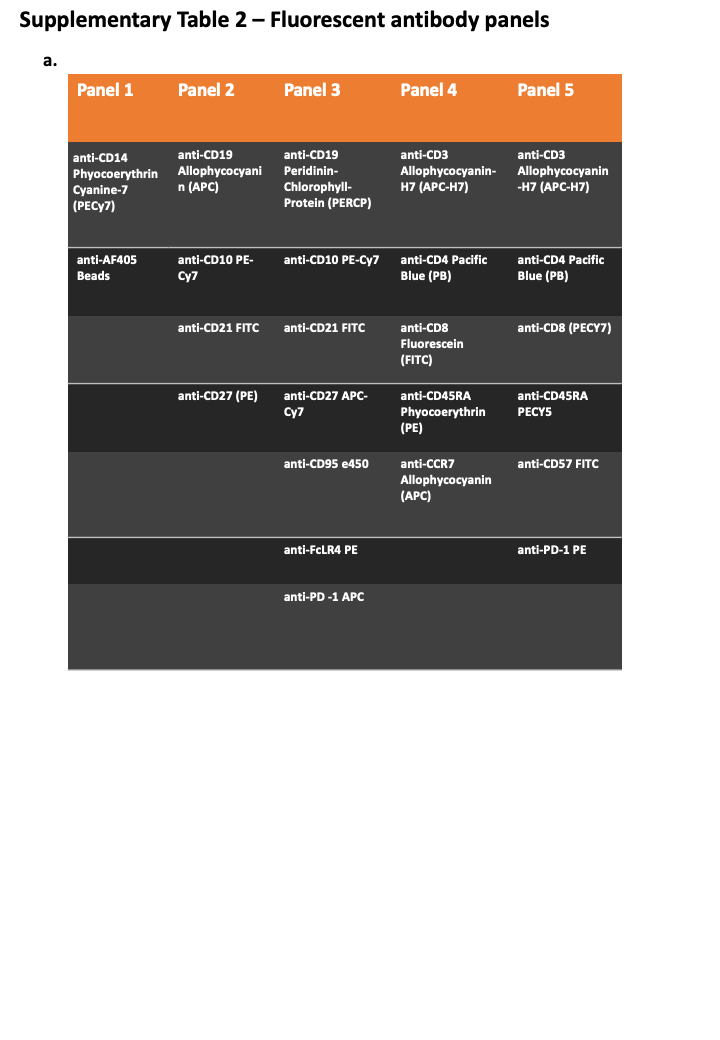

Supplement: jiac133_Supplementary_Data [file jiac133_supplementary_data.zip › Slide15.tiff]

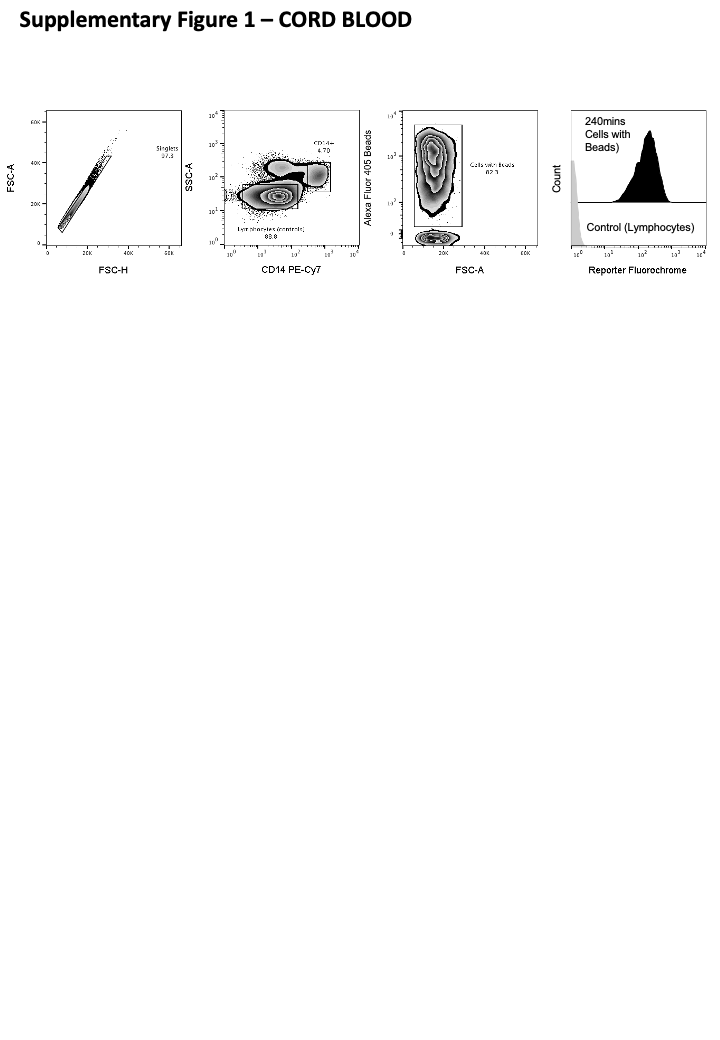

Supplement: jiac133_Supplementary_Data [file jiac133_supplementary_data.zip › Slide6.tiff]

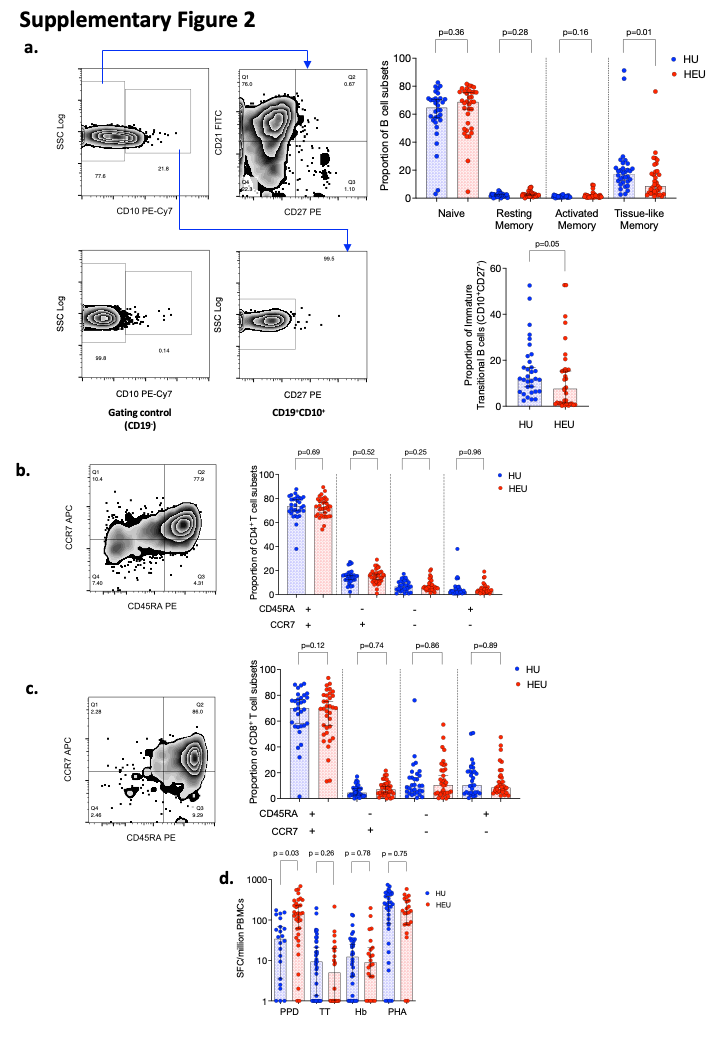

Supplement: jiac133_Supplementary_Data [file jiac133_supplementary_data.zip › Slide7.tiff]

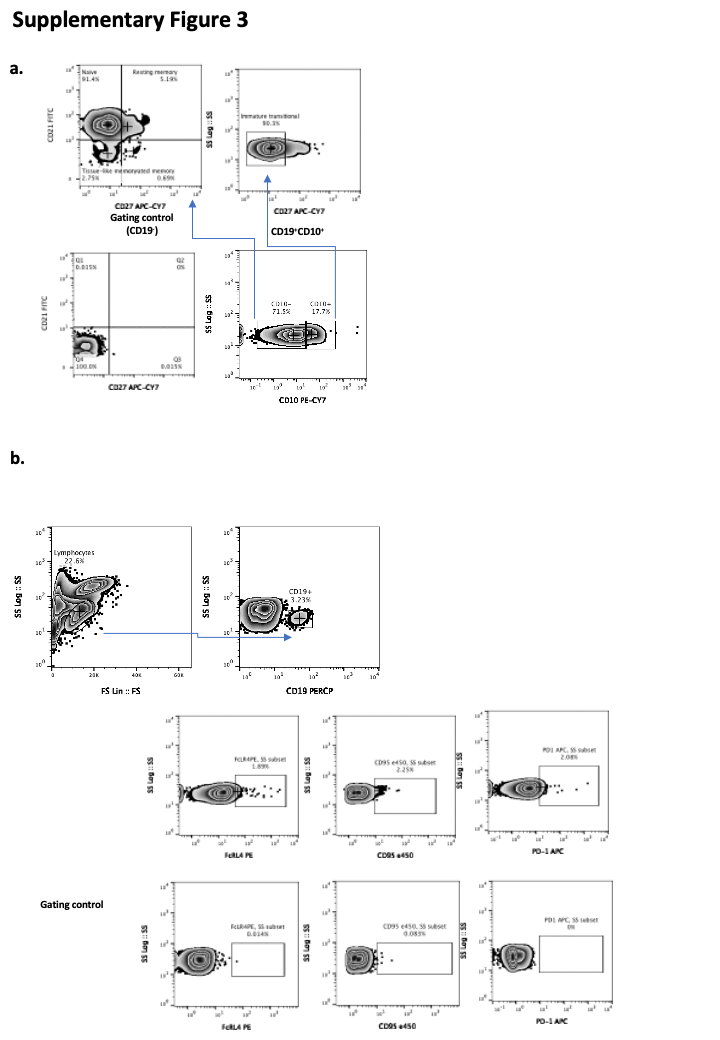

Supplement: jiac133_Supplementary_Data [file jiac133_supplementary_data.zip › Slide8.tiff]

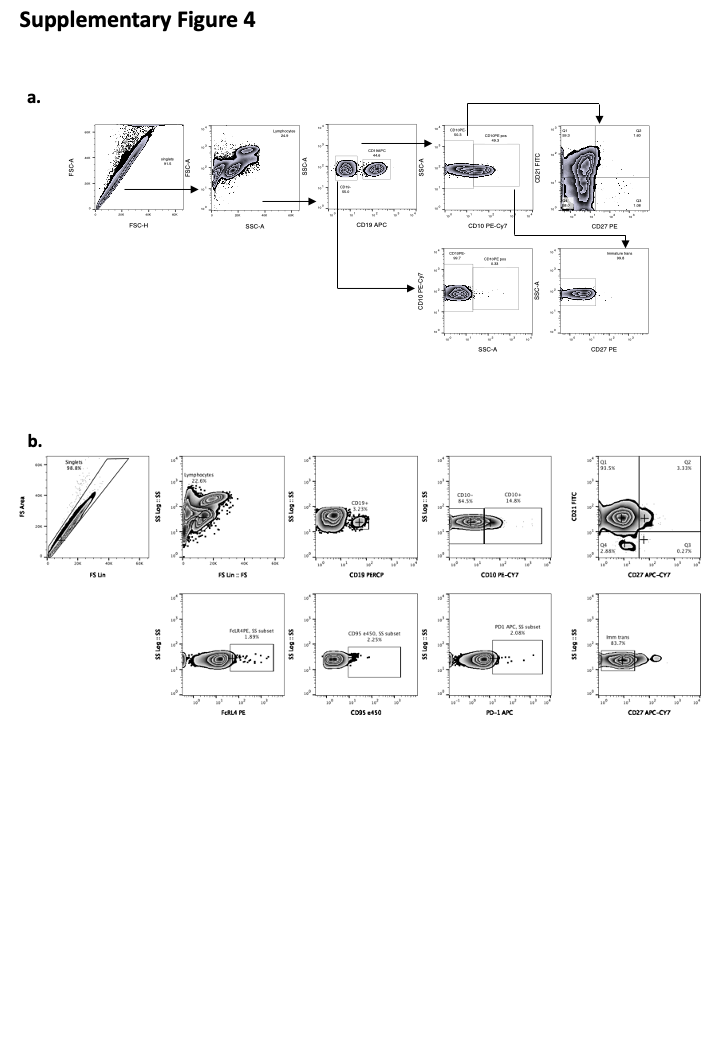

Supplement: jiac133_Supplementary_Data [file jiac133_supplementary_data.zip › Slide9.tiff]
